# Acute-Onset Achalasia Following a Recent COVID-19 Infection: A Case Report

**DOI:** 10.7759/cureus.38803

**Published:** 2023-05-09

**Authors:** Asad Ullah Wasim, Muhammad Wasim Khan, Osama A Khan, Kholoud Soliman Almatraf

**Affiliations:** 1 Internal Medicine, Air University - Fazaia Medical College, Islamabad, PAK; 2 Division of Clinical and Translational Research, Larkin Community Hospital, Miami, USA; 3 Internal Medicine, Al Iman General Hospital, Riyadh, SAU

**Keywords:** chest ct scan, aperistalsis, esophageal dilation, covid-19, achalasia cardia, achalasia

## Abstract

Achalasia is a rare esophageal motility disorder that leads to dysphagia, regurgitation, and several other symptoms. While the etiology of achalasia is not completely understood, studies have suggested an immune reaction to viral infections, including severe acute respiratory syndrome coronavirus 2 (SARS-CoV-2), as a potential cause. Here, we present a case report of a previously healthy 38-year-old male who presented to the emergency room with severe shortness of breath, recurrent vomiting, and dry cough, that had progressively worsened over five days. The patient was diagnosed with coronavirus disease 2019 (COVID-19), and a chest CT also revealed prominent features of achalasia with a markedly dilated esophagus and areas of narrowing at the distal esophagus. The initial management of the patient included IV fluids, antibiotics, anticholinergics, and corticosteroid inhalers which improved his symptoms. This case report highlights the importance of considering the acute-onset of achalasia in COVID-19 patients and the need for further research on the potential association between SARS-CoV-2 and achalasia.

## Introduction

Achalasia is a motor disorder of the esophagus that occurs due to a loss or reduction of myenteric plexus neurons in the distal esophagus and lower esophageal sphincter (LES), leading to the LES's failure to relax and aperistalsis. The progressive destruction of inhibitory ganglion cells in the distal esophageal wall results in the LES's unmatched excitation and inability to relax, which causes a build-up of food and liquid in the esophagus [1]. Achalasia can cause several symptoms including dysphagia, chest pain, vomiting, regurgitation and pneumonia due to aspiration [2]. With an annual incidence of roughly 1.6 incidents per 100,000 people and a prevalence of 10 cases per 100,000 people, achalasia has been labeled as an uncommon disorder [1]. Despite numerous theories being put forth, the precise cause of achalasia is not completely understood. The proposed etiologies include autoimmune, viral, genetic, and environmental causes [3,4].

Previous studies suggested that an immune reaction to viral infections, such as the herpes zoster and measles viruses, causes the attack on esophageal neurons in achalasia. This attack is mediated by CD3/CD8-positive cytotoxic T lymphocytes, eosinophils, and mast cells [4,5]. Recently, reports have indicated that the severe acute respiratory syndrome coronavirus 2 (SARS-CoV-2) virus also has a similar role in the development of achalasia [6,7]. While the respiratory system is the predominant site of infection for SARS-CoV-2, it is important to note that the virus may infect other organs, including the GI tract. Many coronavirus disease 2019 (COVID-19) patients have reported having GI problems, in fact some studies even suggested that up to 50% of patients may develop GI symptoms such as diarrhea, nausea, and vomiting. The virus enters host cells via the angiotensin-converting enzyme-2 (ACE2) receptor, which is found on several cell types throughout the human body, including the lining of the esophagus. When a virus infects a cell, it multiplies and triggers an inflammatory cytokine response, especially IL-6, which mediates the numerous GI symptoms. In severe cases, cytokine release can also contribute to a systemic inflammatory response, leading to a cytokine storm [8,9].

## Case presentation

A 38-year-old male presented to the emergency room of Al Iman General Hospital, Riyadh with severe shortness of breath and recurrent vomiting associated with a dry cough that had progressively worsened over five days. The patient had no history of fever, abdominal pain, change in bowel habits, recent travel, or contact with a sick patient. The patient was a former smoker who quit smoking a year ago, and for the previous six years, he has worked as a security guard at a stone-crushing factory. He had no prior medical history of any disease.

On examination, the patient was conscious and oriented to time and place, but under distress and unable to speak, with signs of accessory muscles being used. His jugular venous pressure was not raised, and there were no signs of lower limb edema. The patient's vital signs were as follows: heart rate of 100 bpm, blood pressure of 130/80 mmHg, oxygen saturation (SpO2) of 88% at room air, and temperature of 37.7°C. Respiratory examination revealed silent chest on auscultation, which after initial management, started to produce wheezes. Cardiovascular examination showed an S1S2+0 tachycardia. His abdomen was soft and laxed. The visual triage checklist for acute respiratory infection was high, and therefore a COVID-19 test was ordered [10].

**Figure 1:**
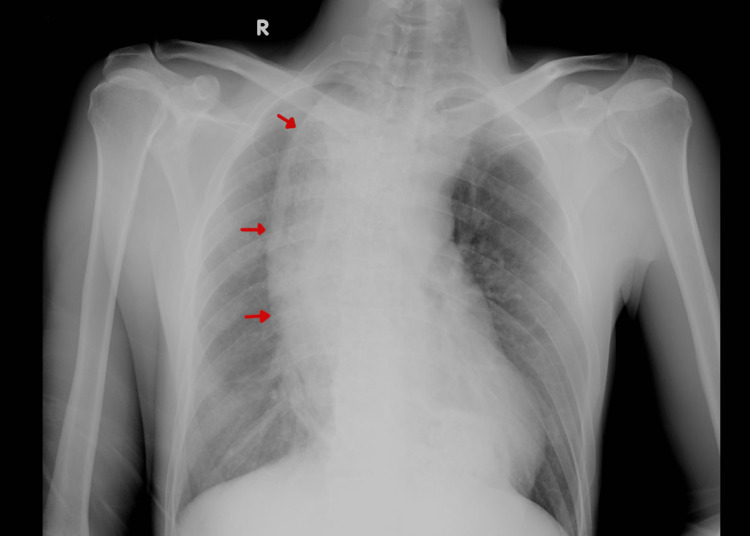
Chest X-ray (anteroposterior view) with red arrows pointing to the noticeably dilated esophagus, which is shown to be smoothly tapering below to the gastroesophageal junction.

A complete workup was done, including a complete blood count, serum biochemistry, arterial blood gases, coagulation profile, an electrocardiogram, and a chest X-ray. The labs showed leukocytosis of about 203/uL with blood gases showing high partial pressure of carbon dioxide (PCO2) of about 80mmHg. The chest X-ray (Figure 1) showed a prominent widening of the mediastinum and right basal pulmonary infiltrates [11]. The patient was then connected to bilevel positive airway pressure (BiPAP) and received initial management of Ventolin, Atrovent, and Pulmicort nebulization along with magnesium sulfate and epinephrine.

Management

**Figure 2:**
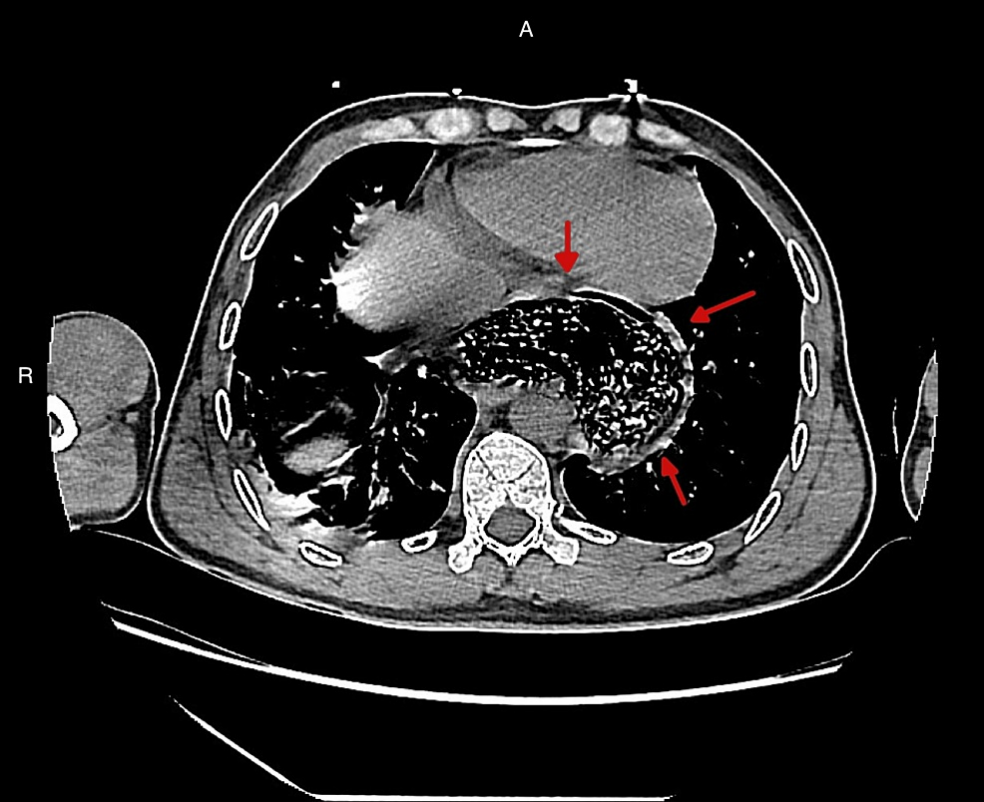
Transaxial chest CT without contrast. There is a severely dilated esophagus (red arrows) and a significant amount of air and residual food debris can be seen.

**Figure 3:**
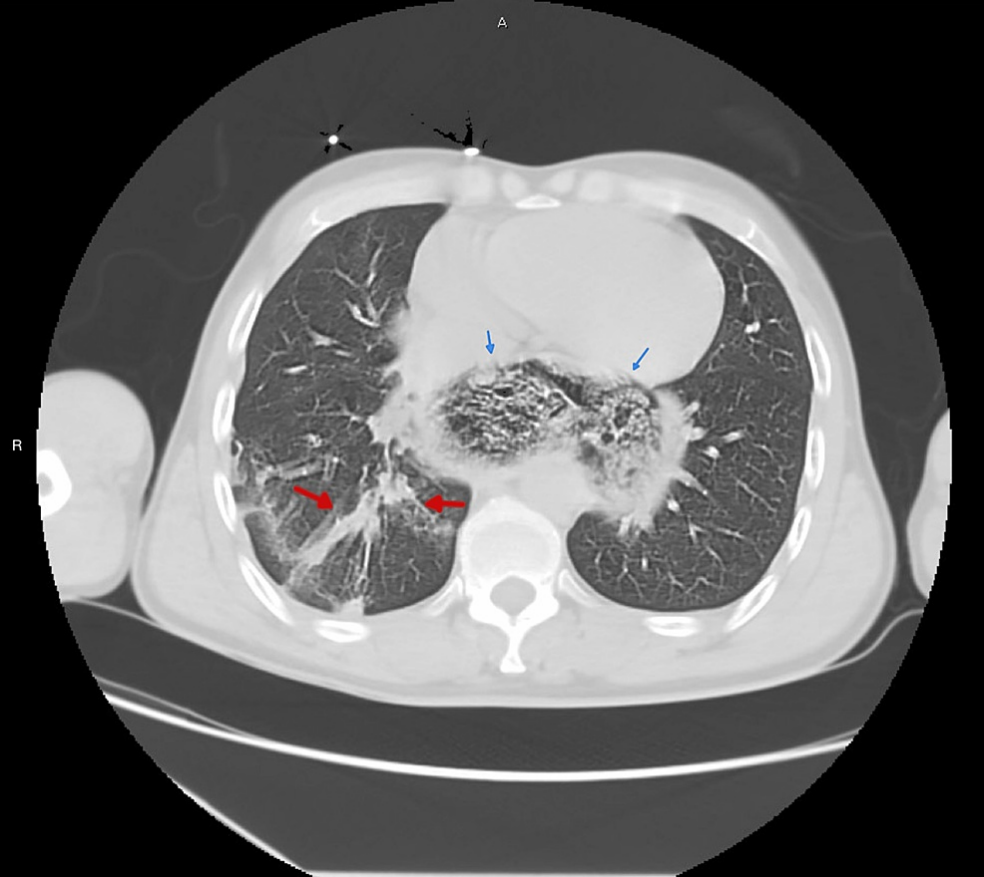
Lung enhancing transaxial CT scan of chest at the level of carina showing linear atelectasis (red arrows) and dilated esophagus with food debris and air (blue arrows).

The patient was admitted to the intensive care unit (ICU) with a provisional diagnosis of bronchopneumonia and suspected COVID-19 infection. In the ICU, he was started on initial management which included IV fluids, antibiotics (ceftriaxone and azithromycin) and prophylactic enoxaparin for the prevention of venous thromboembolism. His inhalers of Atrovant, Ventolin, and Pulmicort were being continued during this time. A computed tomography (CT) of chest was advised to evaluate the mediastinal widening (Figure 2), which revealed features of achalasia, with a markedly dilated esophagus extending above the level of the clavicle and thoracic inlet, with associated areas of narrowing seen at the distal esophagus. The CT report also included that there was a mass effect from the esophagus on the trachea. There were no signs of cardiomegaly or pericardial effusion. There were no radiological findings of aortic dissection, lung masses, or bone lesions. The lungs also showed areas of linear atelectasis seen in the right lower lobe (Figure 3). 

The patient's COVID-19 test result returned positive the following day, which, in conjunction with his five-day history of symptoms, physical exam findings, lab results, and CT imaging, that were indicative of achalasia, provided further evidence of a possible association between SARS-CoV-2 infection and achalasia.

After ICU management, his COVID-19-related pulmonary improved, and he was referred to a gastroenterologist for endoscopic pneumatic dilation to evacuate the esophageal contents. Later he was discharged and recommended to take omeprazole orally, advised to avoid solids and scheduled for follow-up in an outpatient clinic to complete the esophageal manometry test and barium swallow study.

## Discussion

The patient's presentation included severe dyspnea, which initially led to suspicion of COVID-19-related bronchopneumonia due to its common respiratory symptoms. The visual triage checklist for acute respiratory illness, used in Saudi Arabia for rapid evaluation and potential isolation of COVID-19 patients, also supported this suspicion based on the patient's high score [10]. However, the presence of recurrent vomiting after meals and mediastinal widening observed on chest X-ray prompted us to consider an alternative diagnosis. This led to the recommendation of a CT scan which confirmed the diagnosis of achalasia along with the observation of linear atelectasis in the right lower lung lobe. Subsequently, the polymerase chain reaction (PCR) report also confirmed a positive result for COVID-19. Definitive diagnosis of achalasia involves further investigations such as esophageal manometry, which is the gold standard, or barium swallow study, which shows characteristic “bird’s beak” appearance [12,13].

The development of achalasia symptoms can vary from person to person. Some individuals may experience the development of symptoms for years, while others may have a sudden onset of symptoms. According to a review article published in the Deutsches Ärzteblatt International journal, the onset and development of symptoms before the diagnosis can range from several weeks to years [14]. However, it is important to note that the exact timeline can vary depending on the individual and the severity of their condition. Achalasia can also be divided into three different subtypes (type I, II & III) according to the number and severity of symptoms [15]. It is also worth noting that in some cases, achalasia may be asymptomatic and only discovered incidentally during medical imaging or testing for other conditions [16].

Complications of achalasia include further dilation of esophagus, leading to increased food impaction, resulting in progressive dysphagia from solids to liquids. Other complications include esophageal perforation, aspiration and metaplasia followed by esophageal carcinoma [17]. Tracheal compression, like the one in this patient's CT scan, is a relatively uncommon aspect of achalasia, and there aren't many cases with such complications documented in the literature [18]. The treatment options for achalasia include medications, endoscopic therapies, and surgical interventions. Medications such as nitrates and calcium channel blockers can be used to relax the LES and ease symptoms. Endoscopic therapies, such as balloon dilation and botulinum toxin injection, aim to disrupt the LES muscle and allow for easier food passage. Surgery is also an option, with laparoscopic myotomy being the most common procedure, where the LES muscle is cut to improve esophageal emptying. The choice of treatment depends on the severity of symptoms, patient preference, and the experience of the treating physician [19].

In this unique case of acute onset achalasia following a COVID-19 infection, we emphasize the fact that frequent vomiting and regurgitation should not be overlooked as general GI symptoms of COVID-19 infection. Instead, it highlights the need for further imaging to establish a definitive diagnosis, enabling early recognition of the disease and timely treatment to prevent potentially life-threatening complications. 

## Conclusions

This case provides a useful look at a patient presenting with severe dyspnea and recurrent vomiting in the ED following a recent COVID-19 infection who was ultimately diagnosed with achalasia. The patient was a 38-year-old male with no significant medical history, who was conservatively managed with IV fluids, antibiotics, anticholinergic and corticosteroid nebulizers. We anticipate that this case report will stimulate further research into the link between viral infections like COVID-19 and achalasia. By gaining a better understanding of the causes of achalasia, we can enhance the accuracy of clinical diagnosis and treatment, as well as mitigate the complications.
